# Biocontrol Agents: Toolbox for the Screening of Weapons against Mycotoxigenic *Fusarium*

**DOI:** 10.3390/jof7060446

**Published:** 2021-06-03

**Authors:** Lucile Pellan, Cheikh Ahmeth Tidiane Dieye, Noël Durand, Angélique Fontana, Caroline Strub, Sabine Schorr-Galindo

**Affiliations:** 1 Qualisud, Univ Montpellier, Avignon Université, CIRAD, Institut Agro, IRD, Université de La Réunion, 34095 Montpellier, France; pellan.lucile@gmail.com (L.P.); cheikh.dieye@umontpellier.fr (C.A.T.D.); noel.durand@cirad.fr (N.D.); angelique.fontana@umontpellier.fr (A.F.); sabine.galindo@umontpellier.fr (S.S.-G.); 2 CIRAD, UMR Qualisud, 34398 Montpellier, France

**Keywords:** mycotoxins, microbial interaction, *Trichoderma*, *Streptomyces*, *Pythium*

## Abstract

The aim of this study was to develop a set of experiments to screen and decipher the mechanisms of biocontrol agents (BCAs), isolated from commercial formulation, against two major mycotoxigenic fungi in cereals, *Fusarium graminearum* and *Fusarium verticillioides*. These two phytopathogens produce mycotoxins harmful to human and animal health and are responsible for the massive use of pesticides, for the protection of cereals. It is therefore essential to better understand the mechanisms of action of alternative control strategies such as the use of BCAs in order to optimize their applications. The early and late stages of interaction between BCAs and pathogens were investigated from germination of spores to the effects on perithecia (survival form of pathogen). The analysis of antagonist activities of BCAs revealed different strategies of biocontrol where chronological, process combination and specialization aspects of interactions are discussed. *Streptomyces griseoviridis* main strategy is based on antibiosis with the secretion of several compounds with anti-fungal and anti-germination activity, but also a mixture of hydrolytic enzymes to attack pathogens, which compensates for an important deficit in terms of spatial colonization capacity. It has good abilities in terms of nutritional competition. *Trichoderma asperellum* is capable of activating a very wide range of defenses and attacks combining the synthesis of various antifungal compounds (metabolite, enzymes, VOCs), with different targets (spores, mycelium, mycotoxins), and direct action by mycoparasitism and mycophagy. Concerning *Pythium oligandrum*, its efficiency is mainly due to its strong capacity to colonize the environment, with a direct action via microbial predation, stimulation of its reproduction at the contact of pathogens and the reduction of perithecia formation.

## 1. Introduction

At the intersection between the health protection requirement of consumers and producers, economic issues of intensive production, and those related to the environment such as climate changes or limiting our impact on biodiversity, the management of mycotoxigenic fungi are of serious concern [1,2,3]. In cereal crops, the first source of consumer exposure to mycotoxins, *F. verticillioides* (fumonisin producer) and *F. graminearum* (trichothecene producer) infect and colonize the ears during flowering and synthesize toxins in the grains in formation. They have the capability of persisting in the crop debris and constitute an important source of inoculum for the following seasons [4].

The most effective control methods against these toxins must be implemented in the field before harvesting and the transformation of product [5], notably with alternative methods to chemical pesticides such as biocontrol [6,7]. In particular, the use of antagonistic microorganisms that prevent or reduce the spread of plant pathogens was investigated and numerous biocontrol agents have been identified [8]. However, few of them are marketed against *Fusarium* spp. (in Europe, only *Pseudomonas chlororaphis* and *Pythium oligandrum* are available under the name Cerall^®^, Belchim Crop Protection and Polyversum^®^, Biopreparáty/De Sangosse, respectively); many factors still need to be studied to ensure effective implementation [9]. Notably, understanding the mechanisms that govern the interaction of BCAs and pathogens over time is of particular interest [10]. The main mechanisms identified against mycotoxigenic fungus are mostly related to parasitism, antibiosis, biotransformation of mycotoxins or biocompetition [11]. Parasitism is a direct attack of pathogen by the BCA that will degrade or consume the pathogen host until it dies [12]. Antibiosis is attributed to BCAs being able to synthesize secondary antifungal or anti-mycotoxigenic metabolites, such as antibiotics or lytic enzymes [13,14]. The detoxification of mycotoxins includes the degradation, transformation and direct absorption of toxins produced by mycotoxin fungi [6,7,15]. Biocompetition may occur when particular ecological niches are simultaneously occupied by pathogens and BCAs, either in terms of nutrients or space [16,17]. Depending on the conditions and type of BCAs considered, one or more of these interconnected mechanisms may be observed, with variable contributions to the overall reduction of pathogen populations. Each of these modes of action can be characterized at different levels (e.g., antibiosis can be demonstrated by the presence of inhibition halos of the growth of the pathogen in dual culture assay but also by precise identification of the metabolite(s) responsible for this inhibition). They can also be related to each other (e.g., the secretion of degradation enzyme can be one of the components of the overall parasitism strategy, or several independent modes of action can have synergic effects, such as nutritional competition and antibiosis). The complexity of these mechanism and their synergy makes their analysis difficult. In addition, in the majority of pathogen(s) vs. BCAs studies, the major approach is the dual culture assay [18,19,20], which gives a good idea of the impact on growth with a limited investment but does not systematically consider the synthesis of mycotoxins. This aspect is nevertheless essential in the interaction between BCAs and mycotoxigenic pathogens because these toxins are naturally synthesized to allow pathogens to protect themselves from competing or invading organisms and can affect the activity of BCAs [10]. Furthermore, this method does not allow targeted identification of the mechanisms triggered by BCAs, or the activation of mechanisms requiring specific conditions. Hence the need to develop specific experimental devices to identify mode of action, at different stages of pathogen evolution.

With the objective in mind of establishing an initial mapping of the various impact of BCAs against mycotoxigenic fungi, this study provides an overview of the potential time-series biocontrol action of three commercial BCAs. They were selected for their contrasting uses, demonstrated efficiency, and microorganism types (*T. asperellum*, *S. griseoviridis*, *P. oligandrum*). To detect potential new modes of action of this BCAs, a toolbox of experimental devices covering different stages of pathogens evolution was developed. Various effects like (1) impact of BCAs on pathogens germination, (2) mycophagous activity of BCAs on pathogens, (3) BCAs chitinase activities and (4) antimicrobial BCAs VOCs, (5) biotransformation of mycotoxin by BCAs, (6) nutritional competition, (7) spatial competition and (8) impact of BCAs on perithecia production by *F. graminearum* were analyzed. BCA/pathogen selection criteria and descriptions are presented in Section 2 as well as the detailed devices developed and used to explore BCAs interactions with mycotoxigenic fungi and identify key weapons used by the BCAs.

## 2. Materials and Methods

### 2.1. Microorganisms, Culture Media and Mycotoxin Analysis

#### 2.1.1. Fusaria Species

*Fusarium graminearum* strain BRFM 1967 and *Fusarium verticillioides* strain BRFM 2251 (CIRM, University of Aix-Marseille, Marseille, France) were selected for their high respective mycotoxins production. *F. graminearum* was isolated from wheat plant and has a deoxynivalenol (DON/15-ADON) chemotype; *F. verticillioides* was isolated from maize kernels, and has a fumonisins (FB_1_/FB_2_/FB_3_) chemotype. Pure cultures were maintained on potato dextrose agar (PDA; BD Difco, Sparks, MA, USA) at 4 °C under paraffin oil. For spore suspensions production, pathogens were actively grown on PDA at 25 °C for 7 days. For *F. graminearum* dual culture assay against BCAs, CYA or CYB (sucrose 30 g; with or without agar 15 g; yeast extract (Biokar, Beauvais, France) 5 g; K_2_HPO_4_·3H_2_O 1 g; NaNO_3_ 0.3 g; KCl 0.05 g; MgSO_4_·7H_2_O 0.05 g; FeSO_4_·7H_2_O 0.001 g; ZnSO_4_·7H_2_O 0.001 g; CuSO_4_·5H_2_O 0.0005 g; per liter of distilled water, pH 6.3 ± 0.2) was used, and for *F. verticillioides* dual culture assay against BCAs, PDA or PDB (BD Difco, Sparks, MA, USA) was used. Comportment of these *Fusariums* species and selection of media are detailed in a previous study [21].

#### 2.1.2. Commercial Biocontrol Agents (BCAs)

Three commercial biological control agents (BCAs) were chosen for their contrasting characteristics and their ability to inhibit the two mycotoxigenic fungi. Explanations on how BCAs were selected and more information about their characteristics and their effects on growth and mycotoxinogenesis of *Fusarium* pathogen in dual culture assays are available in previous work [21]. All BCAs were isolated from their commercial formulation with a classical microbial isolation protocol and conserved in commercial product aliquots (4 °C) or under spore forms in glycerol solution (15%/*v*:*v*/−80 °C). Pure cultures were maintained on ISP4 (agar 18 g; starch 10g; K_2_HPO_4_·3H_2_O 1g; MgSO_4_·7H_2_O 1 g; (NH_4_)_2_SO_4_ 1 g; CaCO_3_ 1 g; FeSO_4_·7H_2_O 0.001g; MgCl_2_ 0.001 g; ZnSO_4_·7H_2_O 0.001 g; per liter of distilled water), PDA, and V8 juice agar (V8 juice 200 mL (Campbell’s, Camden, NJ, USA); agar 15 g; CaCO_3_ 3 g; per 800 mL of distilled water), respectively, for Mycostop, Xedavir, and Polyversum at 25 °C for 7 days for production of spore suspensions or inoculation plug concerning Polyversum. For the rest of the study, the isolates isolated from commercial formulation were referred to using the following abbreviations: Myco for Mycostop/*S. griseoviridis* (Lallemand Plant Care^®^), Xeda for Xedavir/*T. asperellum* (Xeda International^®^), and Poly for Polyversum/*P. oligandrum* (DeSangosse^®^).

#### 2.1.3. Mycotoxin Analysis

For *F. graminearum* samples, 30 mL of acetonitrile/water/acetic acid (79:20:1, *v*/*v*/*v*) were added. For *F. verticillioides* samples, 50 mL of water/acetic acid (99.5:0.5, *v*/*v*/*v*) were added. Samples were homogenized by mechanical agitation for 20 min. *F. graminearum* samples were preliminarily diluted 1:50 in water/acetic acid (99.5:0.5; mobile phase of analyzer) and filtered with a CA filter (0.45 μm, Carl Roth GmbH, Karlsruhe, Germany); *F. verticillioides* samples were directly filtered.

Mycotoxin detection and quantification were achieved using an Ultra High-Performance Liquid Chromatography (UHPLC, Shimadzu, Tokyo, Japan) coupled with a mass spectrometer (8040, Shimadzu, Tokyo, Japan). LC separation was performed using a Phenomenex Kinetex XB Column C18 (50 mm × 2 mm; 2.6 μm particles) at 50 °C, with an injection volume of 50 μL. The mobile phase consists of 0.5% acetic acid in ultra-pure water (A) and 0.5% acetic acid in isopropanol (B) (HPLC MS grade, Sigma, St Louis, MO, USA). Binary linear gradient elution program, for mobile phase A, started with 98%, then 90% at 0.01 min, 45% at 1.5 min, 15% at 3.5 min, 20% at 4 min and 98% at 4.01 min until the end of the elution (11 min). The flow rate was 0.4 mL.min^−1^. The mass spectrometer was operated in electrospray positive (ESI+) and negative (ESI–) ionization mode, and two multiple reaction monitoring (MRM) transitions for each analyte were monitored for quantification (Q) and qualification (q) (Table 1 and Table 2). All data were analyzed using LabSolution Software (v5.91/2017, Shimadzu, Tokyo, Japan, 2017). Limits of detection or quantification (LOD/LOQ in ng mL^−1^, respectively) for each mycotoxin were DON (4/14), 15-ADON (10/35), FB1 (0.03/0.1), and FB2 (0.01/0.05).

### 2.2. Toolbox

#### 2.2.1. Impact of BCAs on Pathogen Germination

Cell-free-extracts (CFEs) of BCAs were prepared as follows. Liquid culture of all BCAs in CYB and PDB was incubated at 25 °C for 10 days at 175 RPM (Unitron INFORS, Bottmingen, Switzerland). The microbial culture was centrifugated (10,000 RPM—4°C—10min; Centrifuge 5804 R, Eppendorf, Hamburg, Germany) and filtered with sterile PES filter (0.22 µm, Sartorius, Göttingen, Germany) to remove filaments and spores produced by BCAs and to sterilize CFEs. Spore suspensions of two BCAs Myco and Xeda were prepared from solid medium as pure cultures in sterile water and filtered with carded cotton and standardized at 1 × 10^5^ spores. mL^−1^. Spore suspensions of pathogen were prepared (1 × 10^4^ spores.mL^−1^) in the same way. In microtube, 250 µL of spore suspensions or CFEs of BCAs were mixed with 250 µL of pathogens spore suspensions and incubated for 30 h. Microscopic observations and counts of germinated spores were conducted on sample of 10µL, every 3–12 h depending on germination speed of pathogens with a Zeiss PrimoStar microscope. The images were taken using a Zeiss Axiocam ERc5s camera (Carl Zeiss Microscopy, Thornwood, NY, USA). For BCA spore suspensions modality, H_2_O was used as control and for BCAs CFEs modality, PDB and CYB was used as control for *F. graminearum* and *F. verticillioides* spores, respectively. Pathogen spores vs. BCA spores or CFEs mixes and controls was set up in triplicate for each analyzed time point and 200 spores on average were considered per replicate to calculate the percentage of pathogen germinated spores. Average values were used to create percentage of pathogen spore germination curves.

#### 2.2.2. Mycophagous Activity of BCAs on Pathogen

**Figure 1:**
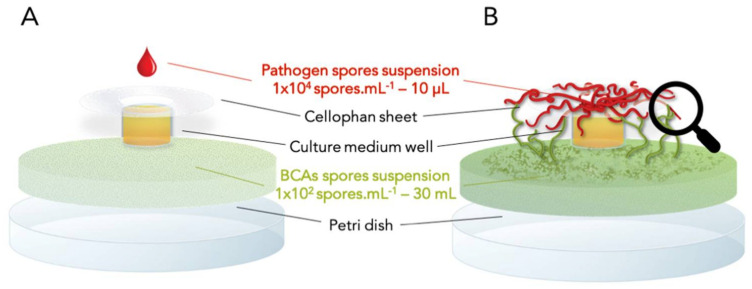
(**A**) Experimental device at beginning of test: in a Petri dish, a washed BCA spore suspension was dropped off around a culture medium well. CYA and PDA were used for *F. graminearum* and *F. verticillioides*, respectively. A cellophane sheet was laid around the well at surface of the BCAs suspension. Pathogen spore suspension was added in culture medium well at the beginning of the experiment; (**B**) experimental device after incubation of 20 days, pathogen was gradually coming out of the well and colonizing BCA suspension thanks to the cellophane sheet support. If BCA present a mycophagous activity, BCA colony could be observed at contact with alive pathogen. Magnifying glass indicates the global position of optical microscopic images.

**Figure 2:**
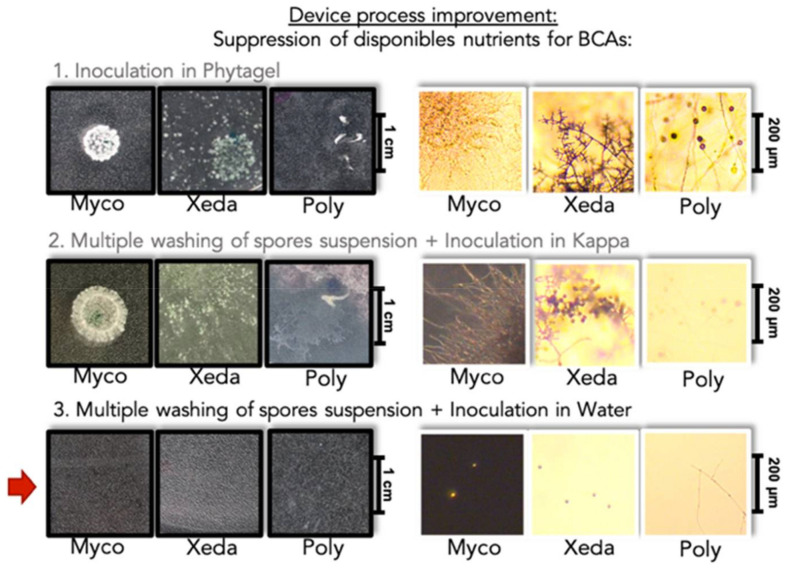
Experimental device process improvement. To ensure that BCAs no longer had sufficient nutrients to grow in the absence of pathogens, various improvements were made based on devices proposed by Balhausen et al. (2015) [22]. All BCAs were directly inoculated on different minimal media without pathogen to verify the absence of growth ((**1**) Phytagel, (**2**) Kappa, (**3**) Water). Optical microscope images framed in black were obtained at macroscopic scale and those framed in white were obtained at microscopic scale after 20 days of incubation. Only (**3**): multiple washing of spore suspension followed by inoculation in water permitted to note the absence of development of BCAs without nutrients. The red arrow indicates that this mode of inoculation has been chosen. Myco: Mycostop^®^, Xeda: Xedavir^®^, Poly: Polyversum^®^.

A specific device was developed, based on and modified from experiments of Balhausen et al. 2015 [22] (Figure 1). A well consisting of a microtube cap (Eppendorf, Hamburg, Germany), containing culture medium was deposited in the center of an empty Petri dish. Pathogen spore suspension was added in culture medium well 48 h before the beginning of the experiment (10 µL—1 × 10^4^ spores.mL^−1^). CYA and PDA were used for *F. graminearum* and *F. verticillioides*, respectively. Then, BCA spore suspensions (30mL—1 × 10^2^ spores.mL^−1^) were dropped off around a culture medium well. To ensure that BCAs had no longer sufficient nutrients to grow in the absence of pathogens, various improvements were made (Figure 2). The main problem was the elimination of nutritional resources for BCAs in media around the well. Different gelling agents were tested: Agar, Phytagel, Kappa, (5 and 3 g.L^−1^). Finally, as all gelling agents tested allowed the growth of BCAs, the spores were only diluted in sterile water. Moreover, the spores were previously washed 5 times to remove any residues from the culture medium used to obtain them. To complete the experimental device, a cellophane sheet was laid around the well at spore suspension surface. After incubation of 20 days at 25 °C, pathogen was gradually coming out of the well and colonizing BCA suspensions thanks to the cellophane sheet support. If BCA presents a mycophagous activity, BCA colony could be observed at contact with alive pathogen. Observations were realized with a Zeiss PrimoStar microscope and pictures were taken using a Zeiss Axiocam ERc5s camera (Carl Zeiss Microscopy, Thornwood, NY, USA). Phase contrast was used only with Mycostop because its spores are very small. Each BCA/pathogen confrontation and controls were set up in triplicate. To validate the results, only modalities where the three triplicates showed growth of BCAs were considered as positive. Control without pathogen (first negative control) and control without BCAs (second negative control) were realized. After 20 days, 10mL of spore suspensions of BCAs were sampled and the mycotoxins produced by pathogens were extracted and quantified as described in previous study (Pellan et al., 2020).

#### 2.2.3. Chitinases Activities of BCAs

In 250 mL Erlenmeyer, 100 mL of minimum liquid medium specific for each BCA was prepared as described in literature: ISP4 liquid for Myco [23], MSM for Xeda [24] and MMPO for Poly [25], supplemented or not (Control) with 1% (w/v) of cell wall powder of *F. graminearum* or *F. verticillioides* [26] (abbreviated G pwd. and V pwd., respectively), i.e., three combinations per BCA. BCAs were inoculated with spore suspensions (5 mL—1 × 10^5^ spores.mL^−1^), except for Poly, which was inoculated with 7-days plugs (5 mm in diameter) in Erlenmeyer flasks and incubated at 25 °C and 150 RPM (Unitron INFORS, Bottmingen, Suisse) for 10 days. Liquid cultures were centrifugated (5 min—10,000 RPM), the supernatant was used as the crude enzymes extract and stored at −20 °C until used to assay enzyme activity. Pellets were used to determinate corresponding dried weight of BCAs, values were used to create BCAs dried weight curves.

Chitinase activity was assessed using a Chitinase assay kit (Sigma-Aldrich, St Louis, MO, USA), according to the manufacturer’s instructions. The kit provides three substrates: 4-Nitrophenyl N,N′-diacetyl-β-D-chitobioside (exochitinase 1 activity detection: chitobiosidase activity), 4-Nitrophenyl N-acetyl-β-D-glucosaminide (exochitinase 2 activity detection: β-N-acetylglucosaminidase activity) and 4-Nitrophenyl β-D-N,N′,N″-triacetylchitotriose (endochitinase activity detection). Cleavage of these substrates release p-nitrophenol which has been quantified at 405 nm by an Enspire Multimode Reader (Perkin Elmer, Waltham, MA, USA). One unit had released 1.0 µmole of p-nitrophenol from the appropriate substrate per minute at pH 4.8 at 37 °C. *Trichoderma viride* chitinase (Sigma-Aldrich, St. Louis, MO, USA) was used as a positive control. Assays were carried out in triplicate, and the results were expressed as mean ± SD (standard deviation). To verify the apparent constitutive synthesis of exochitinase by Poly, supplementary analysis was performed on minimal solid medium.

#### 2.2.4. Antimicrobial BCAs Volatiles Organics Compounds (VOCs)

Petri dishes containing CYA or PDA medium were inoculated in the center with a spore suspension (10 µL—1 × 10^4^ spores. mL^−1^) of *F. graminearum* or *F. verticillioides*, respectively. The three BCAs were inoculated in the center (10 µL—1 × 10^5^ spores. mL^−1^, except for Poly, which was inoculated with 7-days plugs (5 mm in diameter)) in separate plates containing the same media. The lids were removed and two plates containing each one pathogen and one BCA, were placed face to face [26]. The set-up was sealed with a double layer of parafilm (Parafilm M, Amcor, Warmley, UK). A plate containing medium without BCAs was placed face to face with pathogen for the control. The plates were incubated at 25 °C during 8 days. After 3 days then every day of coculture, Petri dishes were photographed, and the colony areas of pathogens were measured in cm^2^ by image analysis using ImageJ software (1.52a, Wayne Rasband National Institute of Health, Bethesda, MD, USA, 2018). Average values were used to create growth curves. At the end of experiment, half a Petri dish of both pathogens was sampled, and the mycotoxins produced by pathogen were extracted and quantified as described in preliminary study [21]. Each BCA/pathogen dual culture and control was set up in triplicate.

#### 2.2.5. Biotransformation of *F. graminearum* Mycotoxins

In order to evaluate the capacity of BCAs to transform, degrade or fix *F. graminearum* mycotoxin (DON), minimum liquid medium (CZB) was inoculated with 7-days plugs (1 cm in diameter) of *F. graminearum* in Erlenmeyer flasks and incubated for 10 days at 25 °C. Liquid cultures were filtered with PES filter (0.22 µm, Sartorius, Göttingen, Germany) to remove mycelium and spores produced by the pathogen and to sterilize medium enriched with mycotoxins. A volume of 10 mL was sampled to quantify the mycotoxin concentration. In parallel, half Petri dishes of medium without mycotoxins (CYA) were inoculated with BCAs or *F. graminearum* alone (control of mycotoxins non-degradation by the pathogen) with 5µL of 1 × 10^5^ spores. mL^−1^, except for Poly, which was inoculated with 7-days plugs (5 mm in diameter) or H_2_O (inoculation control). After 3 days, corresponding to initiation of growth of BCAs, the empty half Petri dishes were completed with enriched mycotoxin medium (CYA with DON content adjusted to 15,000 ng.g^−1^ of media, equating to 100% of mycotoxin content) and incubated for 12 days at 25 °C (same condition as dual culture assays in previous study). Samples were analyzed on days 5, 7 and 12 for all modalities. BCAs colony had gradually colonized the mycotoxin-enriched part. At the end of experiment, in each half of a Petri dish, the remaining mycotoxins were extracted, quantified and summed as in [21], and compared to control plates without microorganisms (mycotoxin content control, 100% of mycotoxin content). The experiment was performed only with the mycotoxins produced by *F. graminearum* because the *F. verticillioides* strain used did not produce enough mycotoxins in liquid medium condition. The main known degradation products resulting from the microbial transformation of mycotoxins were investigated: DOM-1, 3-epi-DON and 3-keto-DON [27,28,29]; particular attention was paid to the identification of peaks that appeared on the chromatogram when mycotoxin amount was reduced, because they could correspond to new product of degradation.

#### 2.2.6. Nutritional Competition between BCAs and Pathogens

Phenotype MicroArrays plates (Biolog, Hayward, CA, USA) measure the carbon consumption profile (PM1), and the nitrogen consumption profile (PM3) of strains for 96 sources and were used according to the manufacturer’s instructions. Carbon sources in PM1 are in the 5–50 mM range and nitrogen sources in PM3 are in the 2–20 mM range. Standardized cell suspensions were prepared in sterile Biolog FF inoculating fluid (transmittance of 81% and 62% at 590 nm for Myco and other microorganisms, respectively), and inoculated into the PMs. PMs were incubated in dark at 25 °C, and growth (optical density) at 750 nm was monitored after 0, 12, 18, 24, 36, 42, 48, 60, 66, 72, 84, 90, 96, 114, and 138 h using an Enspire Multimode Reader (Perkin Elmer, Waltham, MA, USA). Compounds present in wheat or maize were identified through the literature [30,31,32,33], and the consumption of these compounds by different microorganisms were compared to highlight a potential nutritional competition on these natural substrates.

#### 2.2.7. Spatial Competition between BCAs and Pathogens and Potential Antimicrobial Metabolites Diffusion by BCAs

In square Petri dishes, CYA or PDA medium for *F. graminearum* conditions or *F. verticillioides* conditions, respectively, were deposed in nonlimiting quantity (300 mL) representing a total area of 900 cm^2^. In order to identify the most competitive microorganisms between pathogen and BCA, each BCA and pathogen were inoculated alone or in competition (10 µL—1 × 10^4^ spores. mL^−1^, except for Poly, which was inoculated with 7-days plugs (5 mm in diameter)) and incubated for 25 or 40 days at 25 °C, for *F. graminearum* test or *F. verticillioides* test, respectively (i.e., until full coverage of the available space). During the test, Petri dishes were photographed and the colony area of pathogens and BCAs were measured in cm^2^ by image analysis using ImageJ software (1.52a, Wayne Rasband National Institute of Health, Bethesda, MD, USA, 2018). Average values were used to create growth curves. Particular attention was paid to the contact time and potential overgrowth of microorganisms on each other.

#### 2.2.8. Impact of BCAs on Perithecia Production of *F. graminearum*

The production of perithecia was set up based on existing protocol [34,35,36]. *F. graminearum* was inoculated on solid Carrot agar medium and incubated at 22 °C. When the mycelium had covered the whole area, its aerial part was scraped off with a sterile spreader. After 24 h, the same process was repeated. Then, TWEEN20^®^ at 2.5% (*v*:*v*) was supplemented with BCA spores (1 × 10^4^ spores. mL^−1^), except Poly which was added with microfilaments. A volume of 1 mL of this spore’s suspension with TWEEN20^®^ was deposed and spread in a Petri dish, inducing stress and leading to the formation of perithecia. TWEEN20^®^ at 2.5% (*v*:*v*) was used as control.

#### 2.2.9. Data Expression and Statistical Analysis

Statistical data analysis was achieved with R Software (3.4.4, R Foundation for Statistical Computing, Vienna, Austria, 2017). Normality and homogeneity of variances were verified with the Shapiro–Wilk test (with Holm–Bonferroni correction) and Levene’s test, respectively. For each pathogen, the effect of BCAs treatments was tested with a one-way ANOVA and multiple comparisons of means were done with Tukey’s test (α = 0.05). To consider complete evolution of interaction, and not only a final point, the areas under the curves were calculated for the germination test, the evolution of weight during chitinase test, the evolution of mycelial area during COV test, the spatial and nutritional competition test. For the visualization of specific differences between microorganisms nutrient profiling, the negative control was subtracted and a hierarchical classification and clusterization was carried out with the FactoMineR package. Absorbance values of the negative control were subtracted from the measured values for the different modalities.

## 3. Results

### 3.1. Impact of BCAs on Pathogens Germination

**Figure 3:**
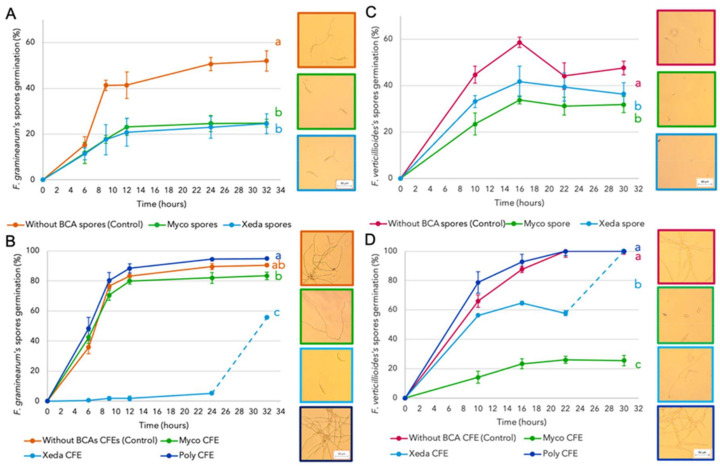
Effect of BCAs on germination of pathogenic spores. *F. graminearum* spores: (**A**) in contact with BCA spores and (**B**) in contact with BCA cell-free-extracts; on germination of *F. verticillioides* spores: (**C**) in contact with BCA spores and (**D**) in contact with BCA cell-free-extracts. Optical microscope images were taken after 24 h of contact between pathogen spores and BCA spores or CFEs at 25 °C. Part of the curve in the dotted line indicates an increase of pathogen spore’s germination percentage but with reduced length germ tubes. CFE: cell-free-extract; Myco: Mycostop^®^, Xeda: Xedavir^®^, Poly: Polyversum^®^. ANOVA test independent for each competition modality on area under the growth curves (AUGCs), *p*-value < 0.05.

During germination test, spores and cell-free-extracts (CFEs) of BCAs were put in contact with spores of two pathogens *F. graminearum* and *F. verticillioides*. The evolution of coculture was observed to identify the influence of BCAs on the earliest stage of pathogen cycle: the spore germination (Figure 3). Concerning the direct contact between spores of BCAs and macroconidia of *F. graminearum* (Figure 3A), the two BCAs reduce the germination of pathogen throughout the assay with an inhibition of pathogen germinated spores of 50% and 53% for Myco and Xeda, respectively. *F. verticillioides* spore germination is less affected by contact with BCA spores (39% and 22% of inhibition by Myco and Xeda, respectively) (Figure 3C). Concerning the contact between CFEs of BCAs and pathogen spores, the germinative ratio of control was higher probably due to higher nutrient availability in liquid medium dedicated to the CFEs. Despite this higher ratio of the control, a more contrasted effect could be observed. Xeda’s CFE has stronger effect against germination of *F. graminearum* (82% of inhibition, Figure 3B). After 24h of contact, with almost total inhibition of germination, an increase of pathogen germinated spores could be observed, but the germ tubes remained restricted in size (50–70 µm on average). For *F. verticillioides*, Myco’s CFE had more influence on spore germination (76% of inhibition, Figure 3D), with atrophied germ tubes and this effect was maintained over time. No effect of CFEs from the Poly culture could be detected on the percentage of germination of pathogenic spores, or even stimulation and the differences with controls were not significant. Nevertheless, length and branching of pathogenic germ tubes in contact with Poly’s CFE was more important than control.

### 3.2. Mycophagous Activity of BCAs on Pathogens

**Figure 4:**
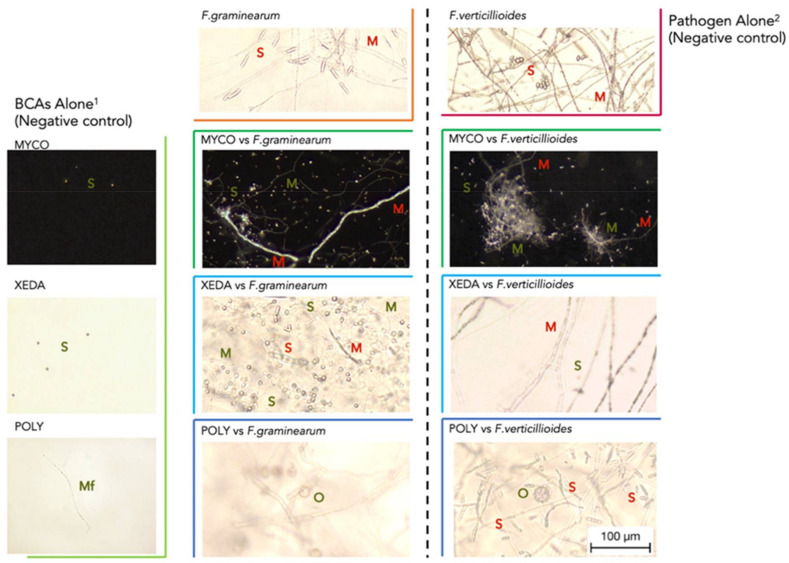
BCAs mycophagous activity against pathogens. Optical microscope images were taken after 20 days of contact between BCA spore suspensions and live mycelia of pathogens at 25 °C. ^1^BCAs alone: devices were inoculated with spore suspension only, and the pathogen spore suspension was replaced with water in the culture well. ^2^Pathogens alone: devices were inoculated only with the pathogen spore suspension in the culture well, the BCA suspension was replaced with water. S: spores, M: mycelium, Mf: micro-filament, O: oospores, in green or red for BCA and pathogen, respectively. Myco: Mycostop^®^, Xeda: Xedavir^®^, Poly: Polyversum^®^.

At the beginning of contact between live mycelium of pathogen and spores of BCAs in developed device to reveal mycophagous activity, contrasted observations could be realized (Figure 4). In absence of pathogen, BCAs were unable to develop, but the presence of nearby living mycelium of pathogens allowed the detection of potential mycophagous activity in the coculture system.

Myco was able to grow in contact with the pathogen *F. graminearum*. Spores, produced in large quantities compared to Myco alone control, were accumulated along the pathogenic hyphae. They formed very fine filaments that were able to bind to the pathogen. Myco also had the ability to proliferate in contact with *F. verticillioides* mycelium, even with a greater development of its mycelium/pseudo-mycelium. This one appeared as early as day 10 and formed a pellet that enclosed the pathogen filaments. Xeda was also able to grow directly around the spores or filament of *F. graminearum*. Colonies of Xeda were frequently observed around deformed *F. graminearum*’s spores or filaments. This colonization allowed the production of newly generated Xeda’s spores who then could form colonies of their own. This ability could be considered early as it was clearly evident on day 6. Nevertheless, no germination of Xeda spores could be observed in contact with *F. verticillioides*. A reduction of quantity of pathogen spores could be observed with Poly. The action was similar on the two pathogens with a high proliferation of filaments of Poly from day 3. This BCA had progressively invaded the pathogenic mycelium and characteristic reproduction structures, oogonia, could be observed. After 15 days, mature oogonia containing oospores were starting to come off.

**Figure 5:**
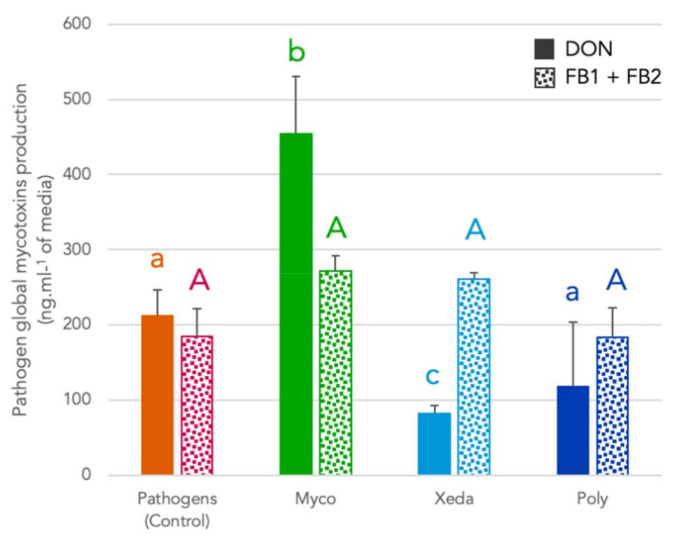
Effect of BCAs mycophagous activity against global production of major mycotoxins of pathogen. Results were obtained after extraction and quantification of mycotoxins in suspension around well, at the end of mycophagy test (after 20 days at 25 °C). Different colors indicate treatment and different patterns indicate mycotoxins type: *F. graminearum*, DON (full colors) and *F. verticillioides*, FB_1_ + FB_2_ (small squares pattern). Myco: Mycostop^®^, Xeda: Xedavir^®^, Poly: Polyversum^®^. ANOVA test for DON (**a**, **b**, **c**) and FB_1_ (**A**), *p*-value < 0.05.

At the end of experiment (20 days), concentrations of mycotoxins diffused on BCAs suspensions were quantified (Figure 5). Although the nutritional conditions of BCAs were very restricted, Xeda was able to reduce DON concentration by 62% compared with control. On the contrary, the presence of Myco strongly stimulated the production of DON by *F. graminearum*. No BCAs allowed the inhibition of FB_1_ diffusion of *F. verticillioides* in these conditions.

### 3.3. BCAs Chitinases Activities

**Figure 6:**
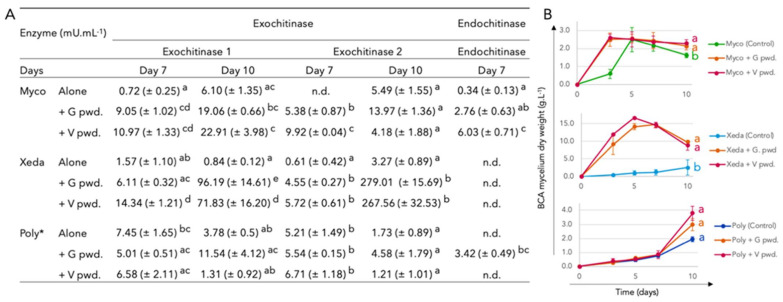
(**A**) BCAs chitinases activities in presence of pathogens mycelium fragments. Chitinase activity (mU.mL^−1^) of BCAs alone or in contact with mycelium powder of pathogen at day 7 or 10 (endochitinase was tested only at day 7). (**B**) Dry weight evolution of BCA during chitinase test (g.L^−1^). Minimum media was liquid and adapted for each BCAs growth. Cultures were carried out at 25 °C under agitation of 150 RPM during 10 days. Exochitinase 1: chitobiosidase activity, Exochitinase 2: β-N-acetylglucosaminidase activity and Endochitinase: endochitinase activity; G pwd.: *F. graminearum* mycelium powder. V pwd.: *F. verticillioides* mycelium powder; n.d.: not detected. * To confirm the apparent constitutive synthesis of exochitinase by Poly, supplementary analysis was performed on solid media, and comparable profile was detected. Myco: Mycostop^®^, Xeda: Xedavir^®^, Poly: Polyversum^®^. ANOVA test, between BCAs (all conditions combined (Alone, + G.pwd., + V pwd.)) for each chitinase (Exochitinase 1, Exochitinase 2, Endochitinase) and day (day 7 or 10) analyzed independently (a, b, c, d), *p*-value < 0.05.

During the growth phase of the microorganisms, the ability of BCAs to produce enzymes to degrade pathogen walls was tested, especially chitinases. The results showed how BCAs modulate this activity and their growth according to the presence or absence of pathogens (Figure 6). On day 7, the presence of the inert pathogen powders stimulated the enzyme activities of exochitinase 1 and exochitinase 2 by Myco, with a 13-fold increase over the control on average of activity of exochitinase 1 and exochitinase 2 (Figure 6A). Xeda had a similar profile with stimulation of exochitinase 1 by *F. verticillioides* powder (9-fold increase) and exochitinase 2 by powders of both pathogens (8.5-fold increase on average). Myco had the additional ability to produce endochitinases, particularly in the presence of the wall cells of *F. verticillioides*. However, the two BCAs had contrasting growth behavior (Figure 6B). After a short delay (5 days), no significant difference of Myco growth between the modalities with and without the addition of pathogen powders was observed. On the contrary, the growth of Xeda was particularly stimulated by the presence of pathogens (10-fold increase on average). The enzymatic activity of Poly did not seem to be affected by the presence of pathogens, but it showed the ability to produce the three tested chitinases. Its growth was not modulated by pathogenic fungal wall cells.

After 10 days, Myco’s chitinolytic activities for exochitinases 1 and 2 were stabilized at around 16 and 8 mU.mL-1 on average of all modalities, respectively (Figure 6A). For Xeda, enzyme activities exploded in the presence of pathogenic powders (100-fold for exochitinase 1 and 83-fold for exochitinase 2). Poly retains approximately the same activity over time with a small stimulation in the presence of pathogen wall cells of *F. graminearum*. To confirm the apparent constitutive synthesis of exochitinase by Poly, supplementary analysis was performed on solid media, and comparable profile was detected.

### 3.4. BCAs Antimicrobial Volatiles Organic Compounds (VOCs)

**Figure 7:**
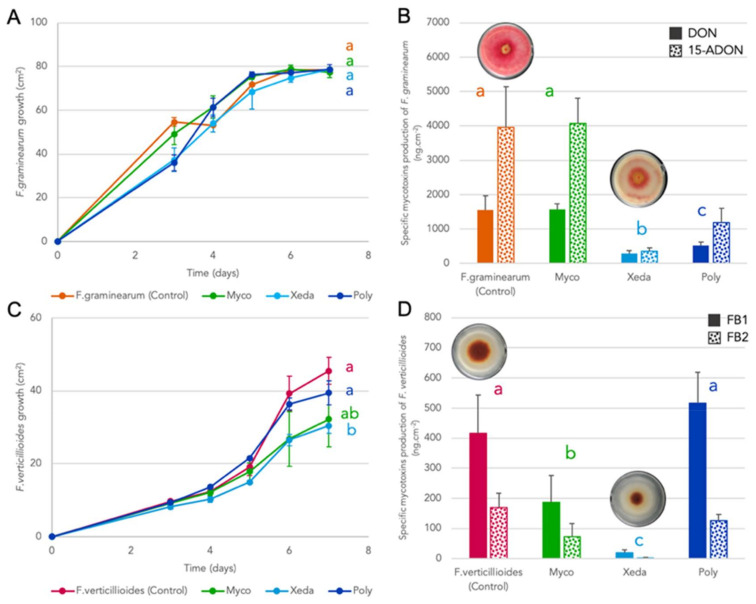
Effect of VOCs from BCAs against growth (**A**,**C**) and mycotoxins production (**B**,**D**) of pathogens. (**A**,**B**) Dual culture of BCA-*F. graminearum* confronted without contact on CYA medium during 8 days (**A**) and at 8 days (**B**) at 25 °C. (**C**,**D**) Dual culture of BCA-*F. verticillioides* confronted without contact on PDA medium during 8 days (**C**) and at 8 days (**D**) at 25 °C. Different colors indicate treatment and different patterns indicate mycotoxins type. Myco: Mycostop^®^, Xeda: Xedavir^®^, Poly: Polyversum^®^. ANOVA test independent for each competition modality on area under the growth curves (AUGCs) (**A**,**C**) or specific mycotoxins production at day 8 (**B**,**D**), *p*-value < 0.05.

BCAs were screened for production of antimicrobial volatile organic compounds against mycotoxigenic *Fusaria* in plates (one turned on top of the other) (Figure 7). Regarding growth effect, only Xeda seemed to produce VOCs able to reduce the growth of *F. verticillioides* by 27% compared to control (Figure 7C). No BCAs influenced the growth of *F. graminearum* (Figure 7A). However, it is interesting to note that important effects were found in the specific production of fungal mycotoxins. *F. graminearum* mycotoxins were affected by Poly COVs (67% and 70% of inhibition of DON and 15ADON, respectively); but especially by Xeda COVs with 82% and 91% of inhibition of DON and 15ADON, respectively (Figure 7B). Myco were able to produce VOCs which can trigger a reduction by half of *F. verticillioides* mycotoxin specific production with 55% and 57% of reduction of FB_1_ and FB_2_, respectively. Xeda treatment again induced the lowest quantities of mycotoxins produced by *F. verticillioides* with nearly complete inhibition (95% and 98% of reduction of FB_1_ and FB_2_, respectively) (Figure 7D). During the test, macroscopic observation of the reverse side of the Petri dish system allowed to observe that the VOCs of Xeda in confrontation against *Fusarium* pathogens caused an important inhibition of the production of pigments by both pathogens.

### 3.5. BCAs Biotransformation of *F. graminearum* Mycotoxins

**Figure 8:**
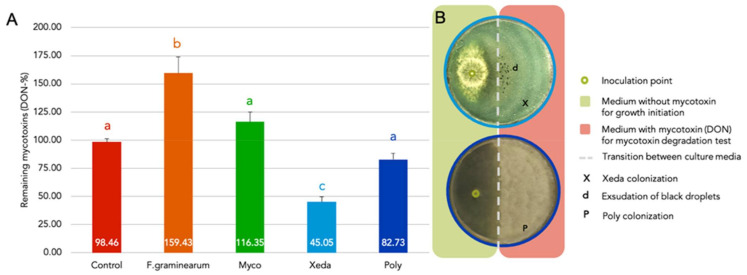
BCAs biotransformation of mycotoxin (DON). (**A**) Remaining mycotoxin of *F. graminearum* (DON) in CYA medium (whole dish extraction), 15,000 ng.g-1 of media = 100%, after 12 days of growth of BCAs at 25 °C. Control corresponded to Petri dish without inoculation of microorganisms. (**B**) Dispositive and visual aspect of Xeda and Poly colonization during biotransformation test. Myco: Mycostop^®^, Xeda: Xedavir^®^, Poly: Polyversum^®^. ANOVA test, *p*-value < 0.05.

During growth phase, BCAs have shown their ability to reduce pathogenic mycotoxin concentrations, even independent of growth reduction as detailed in a previous study [21]. An experimental device was developed to identify the capacity of BCAs to degrade, transform or bind mycotoxins of *F. graminearum*. After 12 days of growth (Figure 8A), it appeared that Xeda is the only one to act directly on mycotoxin reduction with nearly 55% of mycotoxin degraded or transformed (respectively, 13%, 32% and 55% of reduction of mycotoxins after 5, 7 and 12 days of growth). Poly showed a 17% reduction, but this effect was not significant (respectively, 0%, 2% and 17% of reduction of mycotoxins after 5, 7 and 12 days of growth). These two BCAs had a good ability to colonize and cover the enriched medium part of the Petri dish. Black droplets were observed on the Xedavir colony that covered the mycotoxin medium part (Figure 8B). A diffusion of mycotoxins from the enriched medium part to the unenriched part was observed in all plates. However, the presence of Xeda also limited the number of mycotoxins in the free-mycotoxins part of media (compared to control where diffusion was observed), in addition to reduced amount of mycotoxins in the enriched part. In Xeda samples, main DON by-products from microbial transformation known in the literature were searched (DOM-1, 3-epi-DON, 3-keto-DON, [29]). None of these molecules could be detected in the 5-, 7- or 12-day samples despite the significant reductions over time.

Considering the similar condition of biotransformation of mycotoxin test and in vitro dual culture assays detailed in previous study [21], it was possible to hypothesize the importance of mycotoxin biotransformation proportion over global mycotoxin reduction. In order to attempt an explanation, the data of both kinetics of the two BCAs previously considered (Poly and Xeda) were compared. It would appear for Xeda modalities that the percentage of biotransformation of mycotoxin (respectively, 13%, 32% and 56% of reduction of mycotoxins after 5, 7 and 12 days of growth) was an important contribution to global reduction of mycotoxin (respectively, 17%, 64% and 81% of global reduction after 5, 7 and 12 days of growth) and thus as early as the beginning of interaction. On the contrary, regarding Poly modalities, large differences between the results obtained from the global mycotoxin reduction during dual culture assay (respectively, 75%, 92% and 81% of global reduction after 5, 7 and 12 days of growth) and the mycotoxin biotransformation test could be observed.

### 3.6. Nutritional Competition between BCAs and Pathogens

**Figure 9:**
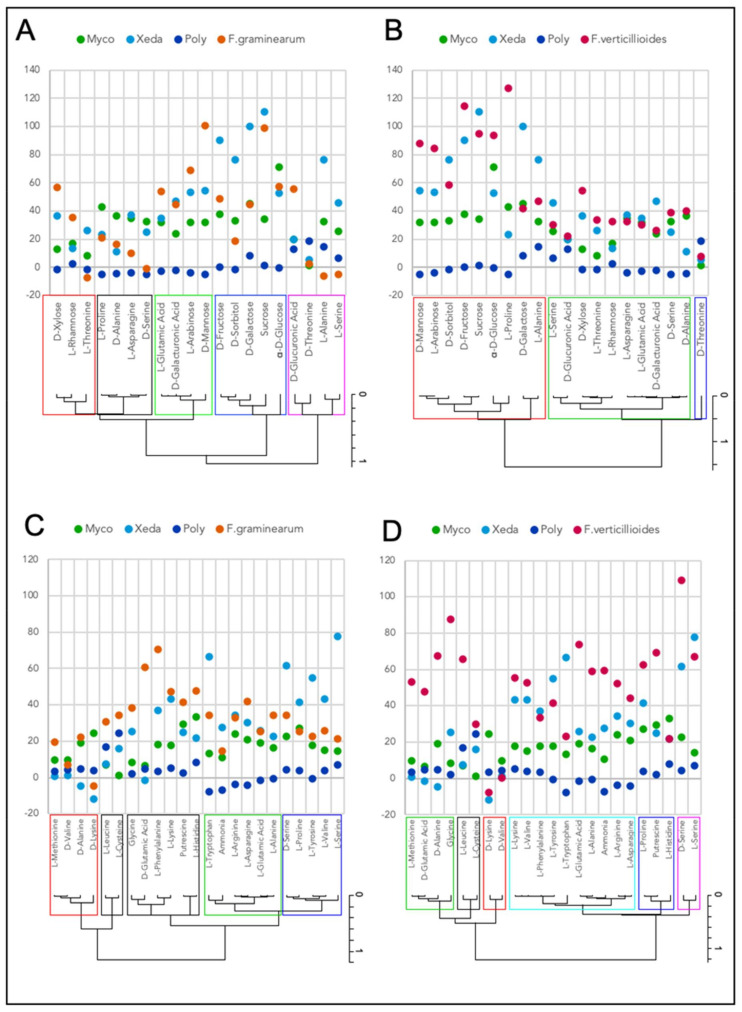
Nutritional profiling of BCAs and *Fusaria* pathogens for cereal compounds (carbon or nitrogen sources). (**A**,**B**) *F. graminearum* and *F. verticillioides*, respectively, vs. all BCAs (Myco, Xeda and Poly) for carbon compounds; (**C**,**D**) *F. graminearum* and *F. verticillioides*, respectively, vs. all BCAs (Myco, Xeda and Poly) for nitrogen compounds. Both panels show optical density measurements over a 138h time course at 25 °C in phenotype micro-arrays (area under growth curve). Major elements found in cereals (wheat and maize for *F. graminearum* and *F. verticillioides*, respectively) are available in literature [30,31,32,33]. Hierarchical classification and clustering were done with all microorganisms independently. Myco: Mycostop^®^, Xeda: Xedavir^®^, Poly: Polyversum^®^.

The capacity of microorganisms to consume different sources of nitrogen and carbon was assessed (Figure 9). A selection of major compounds from wheat and maize was compiled from literature analysis. These compounds were expected to be those for which pathogen and BCA compete in the plant. Some compounds can be found as carbon and nitrogen sources because they have been used either as a source of carbon or as a source of nitrogen (supplemented with glucose).

When comparing the nutrient profiles of BCAs and the phytopathogen *F. graminearum*, the carbon sources were divided into five clusters with 30% of dissimilarity (Figure 9A). The black cluster included compounds averagely consumed and mostly by only Myco, or Xeda and Myco. The green and blue clusters grouped many compounds for which *F. graminearum* and Xeda seemed to compete with high consumption. Increased growth was observed for *F. graminearum* (green cluster, notably arabinose and mannose) and Xeda (blue cluster, mainly sucrose, in direct competition with *F. graminearum*, galactose and sorbitol). The last group of carbon sources contained the best sources for the growth of Poly, with in contrast a low average growth of *F. graminearum* (notably threonine, alanine and serine). For the comparison of the nutrient profiles of BCAs and F. verticillioides, the different nutrient sources were grouped into three clusters (50% of dissimilarity (Figure 9B)). The red cluster grouped compounds that are highly consumed by all microorganisms except Poly. In this cluster, mainly *F. verticillioides* and Xeda were in competition, with once again a stronger growth of Xeda with sucrose (also widely consumed by *F. verticillioides*), galactose and sorbitol. The green cluster included moderately consumed carbon sources, with a branch of compounds mainly to the advantage of *F. verticillioides* and one branch of compounds mainly to the advantage of Myco and/or Xeda BCAs. Threonine remains the compound which alone most stimulates the growth of Poly compared to other compounds and microorganisms (blue cluster). The *Fusarium* pathogens could have very different nutritional patterns. For example, proline strongly stimulated the growth of *F. verticillioides* but less the growth of *F. graminearum*, or the opposite with glucuronic acid.

The same analysis was performed on the nutrient profiles of microorganisms in the presence of different nitrogen sources (Figure 9C,D). When comparing the growth of BCAs and *F. graminearum* (Figure 9C), some groups of compounds were on average more strongly assimilated by BCAs, Myco and Xeda, and the pathogen (pink, green and blue clusters, 40% of dissimilarity); in contrast to Poly which has the best capacity in assimilating nitrogen sources belonging to the red and black clusters. The pink cluster contained the compounds that strongly stimulated the growth of *F. graminearum* in contrast to the blue cluster that contained the sources that stimulated the growth of Xeda. Figure 9D shows the comparison of the consumption profiles of different nitrogen sources according to microorganisms (BCAs and *F. verticillioides*), and few similar patterns were observed (20% of dissimilarity between clusters). Globally the first two groups show compounds highly consumed by *F. verticillioides* (green and black) in opposition to the BCAs Myco and Xeda which consume them less. Opposite profile could be observed in the red cluster. The second branch of the dendrogram (yellow, blue and pink group) shows more competition between BCAs and *F. verticillioides* (particularly Xeda, especially on compounds such as phenylalanine, tyrosine or serine). It should be noted that some compounds such as lysine or valine reduced the growth of both pathogens compared to Myco.

### 3.7. Spatial Competition between BCAs and Pathogens

**Figure 10:**
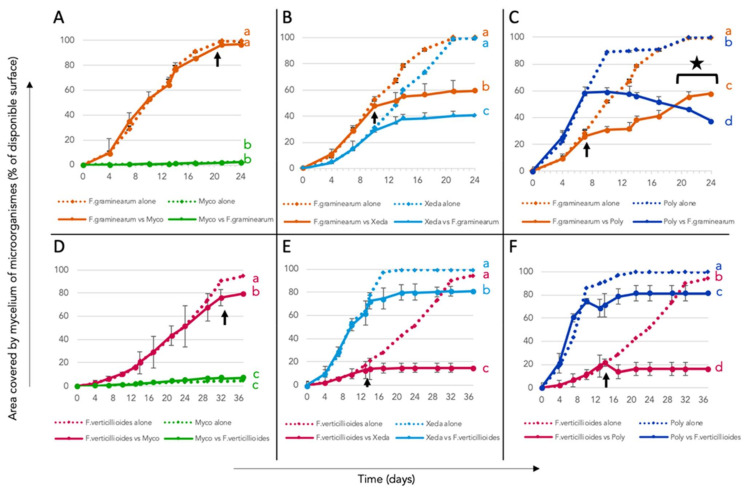
Spatial competition between BCAs and pathogens. (**A**–**C**): CYA medium nonlimiting with *F. graminearum* in competition against Myco, Xeda and Poly, respectively, or each one alone, during 25 days at 25 °C. (**D**–**F**) PDA medium nonlimiting with *F. verticillioides* in competition against Myco, Xeda and Poly, respectively, or each one alone, during 40 days at 25 °C. Arrow indicates first contact between BCA and pathogen and star indicates overgrowth of BCA on pathogen colony; on confrontation curves. Myco: Mycostop^®^, Xeda: Xedavir^®^, Poly: Polyversum^®^. ANOVA test independent for each competition modality on area under the growth curves (AUGCs), *p*-value < 0.05.

By providing more time and space for the microorganisms to grow, spatial competition between BCAs and pathogens was tested in nonlimiting nutritional conditions and contrasted profiles could be observed (Figure 10). In competition with *F. graminearum*, Myco is widely outdistanced in terms of its ability to colonize the environment, alone or in association with the pathogen (Figure 10A). The pathogen outperformed Xeda. However, this BCA allowed a significant reduction of 30% in the progression of the pathogen from the contact point (day 10, Figure 10B). Poly was able to colonize the environment faster than *F. graminearum* whether alone or in confrontation. It also allowed a significant reduction in pathogen development from the point of contact (−45%). At the end of kinetics (20 days), Poly continued to progress over the pathogen’s mycelium (Figure 10C). In competition with *F. verticillioides*, Myco was overtaken in spatial competition. However, a slight inhibition of the pathogen before contact during coculture was observed (Figure 10D). Xeda and Poly were able to grow faster than the pathogen and won the spatial competition in both cases (alone or confronted to pathogen). When cocultured, they reduced pathogen colonization by 70% and 67%, respectively, Figure 10D,E). Overall, the spatial competition induced by BCAs was more effective on *F. verticillioides* than on *F. graminearum*.

### 3.8. Perithecia Production

**Figure 11:**
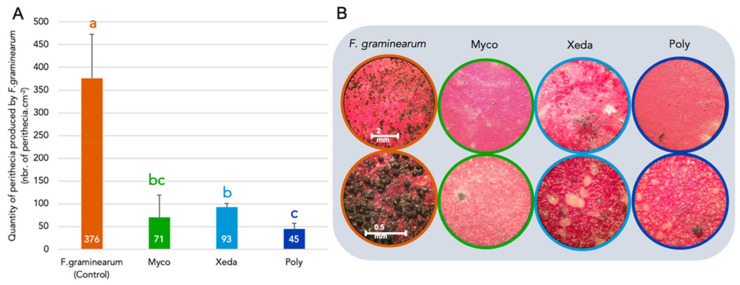
Effect of BCAs on perithecia production of *F. graminearum*. A. Quantity of perithecia produced by *F. graminearum* on carrot agar after 6 days of contact with BCAs at 22 °C, B. Visual aspect of treated or control perithecia test, microscopic images were taken with binocular loupe (×20 and ×150 enlargement). Myco: Mycostop^®^, Xeda: Xedavir^®^, Poly: Polyversum^®^. ANOVA test, *p*-value < 0.05.

At the end of its life cycle, *F. graminearum*, produces dark morphological structures with ascospores enclosed, called perithecia, to assure its conservation. The potential of BCAs to inhibit the synthesis of perithecia was evaluated (Figure 11). *F. graminearum* (Control) had produced many perithecia, averaging 0.08 mm in diameter, but all three BCAs strongly inhibited the formation of its survival structures. Myco caused a slight re-emergence of pathogenic filaments that did not change into perithecia but were tangled with its small colonies It strongly inhibited the production of perithecia, with an average reduction of 81%, and those that did form were quickly covered with thin Myco filaments. Xeda showed a 75% inhibition of perithecia with a reduction of their development, and a large decrease in the size of perithecia formed. Poly showed the strongest impact with 88% fewer perithecia, the oomycete covered the perithecia from the beginning of their formation and prevented their evolution. Moreover, the small number of formed structures was reduced in size.

## 4. Discussion

Many studies on biocontrol by microorganisms provide a good overview of the different mechanisms that can be implemented by BCAs to prevent or stop the progression of pathogens. It appears that biocontrol is the result of many different types of interactions between microorganisms in a complex synergy [37,38]. In general, studies focus on the characterization of mechanisms occurring in different experimental situations and at different stage of pathogen life cycle [39,40,41,42]. In all cases, pathogens are inhibited by the presence and activities of other microorganisms they encounter. However, to our knowledge, none of these studies follow the different mechanisms concomitantly or not, throughout the entire duration of life cycle interaction (from spore germination to pathogen survival) between different types of BCAs and mycotoxigenic pathogens and under the same experimental conditions. This work can be considered as a complementary approach to studies on precise targeted mechanisms involving the characterization of specific genes or molecules, by providing an integrative view of the interaction [43,44].

### 4.1. Impact of BCAs on Pathogen Germination

The first contact between BCAs and pathogens can occur as early as the spore germination phase. In that way, spore germination inhibitors are frequently investigated as potent BCAs [45]. Our results show that the susceptibility of pathogens to the types of anti-germinative compounds can be variable. *F. graminearum* was more sensitive to secretions produced by BCA spores, suggesting a very early action of BCAs. *F. verticillioides* is more sensitive to secondary metabolites produced in CFEs, which implies a prior synthesis of anti-germinative compounds by the BCAs before coming into contact with the pathogenic spores to inhibit their germination. Adhesion phenomena of BCA spores around pathogenic spores could also be envisaged as it is the case of lactic acid bacteria which adhere to yeast [46].

Myco had a strong impact on spore germination of *F. verticillioides* in particular that are in line with the anti-germinative effect of Myco against *F. verticillioides* during microscopic observations of the first phases of the interaction between these two microorganisms [21]. This effect could be linked to the synthesis of compounds inhibiting the germination of plant pathogen’s spores identified with other *Streptomyces* spp., such as fistupyrone or methyl vinyl ketone [47,48]. Xeda had a major impact on the germination of *F. graminearum* spores, and this effect could be related to the synthesis of anti-germinative compounds such as viridin, which has been identified for *Trichoderma* spp. as an inhibitor of spore germination of different pathogens [14]. Another compound secreted by *Trichoderma asperellum*, the 6-pentyl-α-pyrone, has shown a higher effect on the conidia germination and tube elongation of *F. graminearum* than *F. verticillioides* [49].

### 4.2. Mycophagous Activity of BCAs on Pathogen

After this initial contact, the mycophagy test identified BCAs which were able to grow only in the presence of alive and active mycelium of pathogens as source of nutrients. In literature, the mycophagy capacities were investigated mainly in the case of interaction between bacteria and fungus pathogen, notably in Ballhausen et al. and Rudnick et al. studies [22,50], on which our research was based to build the experimental device. When adapting this type of device to study the mycophagous capacity of fungus and oomycete, many adjustments had to be made. Unexpectedly, many microorganisms have the capacity to consume gelling agents composed of complex polymers or very distant from their common consumed compounds (like Phytagel or Kappa). This is why the removal of all external nutrients was a priority in the design of the experiment. In contrast to the few studies that have been conducted on bacterial mycophagy, fungal mycophagy, is very often associated with mycoparasitism. Mycoparasitism is the subject of many research studies [51] and can be defined as the ability of a mycoparasite to directly attack another microorganism and was mainly tested under optimal nutritional conditions [52,53]. Most of them have focused on *Trichoderma* species [54,55,56]. It would be interesting to continue investigations in order to better understand whether the concepts of microbial predation, mycophagy and mycoparasitism can be considered as the same type of BCA strategy or not.

*F. verticillioides* is very sensitive to the mycophagous capacities of Myco, with early development of its colonies during the test (starting from days 4–8). Indeed, bacteria have found niches in the use of nutrients derived from fungi, with nutritional strategies from the consumption of mycelial exudates to endosymbiosis and mycophagia [57]. Some *Streptomyces* strains have shown mycophagous ability against *Aspergillus niger* or *Gigaspora gigantea*, through the use of sterile sand tests or microscopic observation of interactions [58]. Particular attention was paid to the stimulation of the production of mycotoxin DON by *F. graminearum* in the presence of Myco, during the mycophagy test. Microbial or plant interaction can stimulate the mycotoxin production, as it is the case for other pathogens such as *Penicillium* strains [59], or when *F. verticilloides* are in interaction with its host plant [60]. Very few studies have been carried out on the subject, contrary to the impact of abiotic factors, and it seems that this is the first report concerning the stimulation of DON by a biocontrol agent of genus *Streptomyces*. *F. graminearum* is more sensitive to the mycophagous capacities of Xeda, confirmed by the temporal aspect of the observations with development of its colonies very early during the test (from 4–8 days). No mycophagous activity can be detected in interaction with *F. verticillioides*, that can be surprising from an ecological point, because the two species of *Fusarium* are close and microbial predation was defined as nonspecific [61]. However, their growth and mycotoxin production profiles are very different. Xeda, even under limited nutritional conditions, still successfully maintains the inhibition of mycotoxins produced by *F. graminearum*, suggesting the use of dedicated mechanisms. Poly have high colonization capacity that allowed it to rapidly divert nutrients from pathogens in the mycophagy test. In addition, a strong stimulation of the sexual reproduction of this BCA was observed in contact with pathogens. The perception of ergosterols from the pathogen could trigger this phenomenon as sterols have been identified as inducers of sexual reproduction [62,63], and give to Poly a supplementary advantage. *Pythium oligandrum* has already shown mycoparasitic capacities against fungal and oomycete pathogens [64]. The same questions remain regarding the boundary between mycophagy and mycoparasitism

### 4.3. Chitinases Activities of BCAs

During the growth phase of interaction, various weapons were deployed by BCAs to stop the progression of pathogens, including the synthesis of fungal wall degradation enzymes. The capacity of diverse BCAs to produce chitinases in particular is widely known [65]. In our test, the presence of inactivated pathogens in the form of mycelium powder avoids the use of chitin synthetic media and strongly stimulated enzyme activities, because they can be considered as an element of pathogen perception by the BCAs. It might be interesting to introduce this kind of stimulant when formulating BCAs, especially considering that chitin present in mycelium powder can also stimulate plant defenses [66]. Some studies suggest that chitinolytic enzymes could be directly used as a complement to pesticides to increase their efficiency against fungal pathogens, in order to reduce the concentrations required [67]. The simultaneous study of different types of chitinases made it possible to identify the production of enzyme combinations. Endochitinases cleave chitin chains in random locations, generating low molecular weight oligomers. The two exochitinases studied were chitobiosidases which catalyze progressive release of diacetylchitobiose from terminal nonreducing end and N-acetylglucosaminidases which cleaves oligomeric products obtained by endochitinases into monomers of N-acetyl glucosamine, respectively [65]. These different and complementary modes of action allow maximum antifungal efficiency, and synergistic antifungal activity has been observed for endochitinases in association with exochitinase [37,68]. However, very few studies discuss the differential efficiency of exochitinase intra-family. It seemed that some BCAs produced an exochitinase preferentially to the other one, like Myco; while others strongly reversed the ratios between the two exochitinases studied over time, like Xeda which was able to switch between two chitinase activities. During the first contacts with pathogens, Xeda had already demonstrated its ability to dissolve *Fusarium* hyphae through their mycelia [21]. The production of chitinases by *Trichoderma spp.* is widely known, and several genes encoding chitin- and β-glucan-degrading enzymes have been identified in different species [52,69], and can explain this phenomenon. When quantifying the chitinolytic activities of Xeda, exochitinases were massively detected, but not endochitinases, unlike other species of *Trichoderma* able to produce N-acetylglucosaminidases, endochitinases, and chitobiosidases [70]. However, other strains of *T. asperellum* have shown their ability to produce mainly exochitinases but also small amounts of endochitinase, especially in the contact phase but not in the pre-contact or post-contact phase with the pathogen under study [71], which may suggest that chronology plays an important role in the analysis of chitinolytic activities. Another explanation may be related to the repression of chitinase genes by mycotoxigenic Fusaria and Deoxynivalenol, as described for the BCA *Trichoderma atroviride* [43].

In terms of Poly’s chitinolytic activity tests, it was able to produce all the chitinases tested but constitutively at relatively low levels, under all conditions. These observations are in accordance with the results obtained during microscopic observation of the interaction between Poly and *Fusarium* pathogens, where the connections between Poly and pathogenic hyphae were limited in first steps [21]. Despite this, chitinases could play an important role, notably in association with other lytic enzymes to contribute to the mycoparasitism mechanism of this BCA. In molecular study, transcripts encoding cellulases, glucanases, and proteases are produced by *P. oligandrum* which was grown under biocontrol conditions [72,73].

### 4.4. Antimicrobial BCAs Volatiles Organics Compounds (VOCs)

In addition to enzymes, BCAs can also produce volatile antifungal and/or anti-mycotoxigenic compounds. It has been shown that volatile organic compounds (VOCs) from microorganisms can influence the growth of plant pathogenic fungi [74,75]. Most preliminary studies on the effect of VOCs on pathogens are conducted in the same manner as our study [76,77]. Nevertheless, some of the BCAs are able to produce VOCs that inhibit growth and mycotoxins productions (such as Xeda), while others will specifically inhibit mycotoxin production. Without possible contact, it would therefore appear that compounds synthesized by BCAs can have a direct inhibitory action on both pathogen’s mycotoxin biosynthesis pathway. In view of the results, the synthesis of volatile compounds could be a major advantage in the strategy of selected BCAs. However, the identification and analysis of the volatile interactions between BCAs, as well as the biotic and abiotic factors that influence these relationships, remains to be elucidated [78,79].

Xeda has also shown abilities to produce antifungal VOCs, with a very effective inhibition of mycotoxin production by both pathogens. The identification by GC-MS of compounds produced by *Trichoderma* spp., such as 6-pentyl-alpha-pyrone, viridiofungin or dimethyl disulfide [80,81] could explain the observed effects. In addition, severe discolouration of pathogenic colonies was observed. In *Fusarium*, these pigments like naphthoquinone, may be responsible for phytotoxic, insecticidal, antibacterial, and fungicidal effects [82]. Recently, the synthesis of aurofusarin and the red rubrofusarin, two pigments produced by *F. graminearum*, have been directly correlated with the production of mycotoxins, with health toxicity effects when combined with DON [83]. Xeda could therefore directly reduce the toxicity of molecules produced by pathogens, through the synthesis of these VOCs. Poly can also use its ability to produce VOCs, which have a strong impact on the production of DON mycotoxins. Compared to classical dual culture assay, the effect is strong enough to be considered as part of Poly’s overall control strategies against *F. graminearum* [21]. However, no effect on pathogen growth was detected. Few studies on the production and characterization of VOCs produced by *P. oligandrum* have been performed. However, it appears that P*. oligandrum* is capable of producing nonidentified antibiotic and/or antifungal VOCs against different phytopathogens such as *F. oxysporum* or *Phoma medicaginis* [26,84]. Based on our results without contact, this is the first report of VOCs produced by Poly that could have a direct impact on the mycotoxin biosynthesis pathways of *F. graminearum*.

### 4.5. Biotransformation of *F. graminearum* Mycotoxins

After a variable contact time, some BCAs are capable of biotransforming the mycotoxins produced by *F. graminearum* [27]. This mode of action includes degradation, binding and transformation or conjugation of toxins. Detoxification has been identified in Lactic acid bacteria and *Pseudomonas* spp. [85,86], and many studies consider similar the reduction of mycotoxin in dual culture assays and the detoxification and degradation of mycotoxins [87]. In our case, significant reductions in the major mycotoxins were observed in the biotransformation test of toxins previously isolated from the pathogens. The common by-products resulting from the microbial action on these mycotoxins (e.g., DOM-1, 3-epi-DON and 3-keto-DON [29]) were searched by HPLC-MS/MS. However, none of them could be detected. This phenomenon could be explained by the timing of transformation (mycotoxins could be quickly transformed into these compounds and then into simpler molecules before analysis at 5, 7 and 12 days), or mycotoxins could be transformed into new molecules from other mechanisms of biotransformation. In addition, it is important to mention that the test has been designed to be comparable with the results obtained in “conventional” dual assays. Indeed, the chosen mycotoxin concentration as well as all the characteristics of the inoculation and growth have been preserved (position, inoculum concentration, culture medium, temperature, etc.). For this reason, it is possible to estimate the proportion of degradation phenomenon in the overall aspect of mycotoxin reduction by BCAs [21].

Xeda was particularly able to degrade, bind or transform mycotoxin. It was the most effective in the reduction of mycotoxins, which supports the observations made during tests to identify particular mechanisms (as mycophagy or VOCs test). This mode of action also appears to play an important role in global reduction of mycotoxin. Recently, *Trichoderma* strains have shown their ability to detoxify DON via glycosylation, or zearalenone, which supports its interest in detoxification [88,89]. In an unprecedented way, Poly seems to be able to transform the mycotoxins of *F. graminearum*. Despite a strong effect on overall mycotoxin reduction in dual culture assays, the specific degradation activity is weaker and later than other BCAs. These patterns seemed to indicate a less important part of the biotransformation process in the interaction of pathogens. This difference could be explained by the combination of several mechanisms by Poly to reduce the number of mycotoxins: slight detoxification but also direct action on the biosynthesis pathways of mycotoxins. Recently, a study has shown that microorganisms communication in wheat microbiome regulates epigenetic modifiers that in turn control mycotoxin biosynthesis [90].

### 4.6. Nutritional Competition between BCAs and Pathogens

During their growth phase, BCAs can also compete with pathogens for different ecological resources, such as nutrients or space. These mechanisms have been notably identified in yeasts or yeast-like fungi, that use competition for carbon sources or nitrogen sources as a major biocontrol mechanism [91,92]. Moreover, BCAs that possess this ability were more effective in reducing disease development than BCAs with either mycoparasitism or antibiosis as its mechanism [93]. Research on the composition of wheat and corn has allowed the identification of major compounds for which there could potentially be nutritional competition during contact between BCA and the pathogen on the plant. The analysis permits to evaluate the demand of a microorganism for a compound and the potential competition with other microorganisms for this compound. In general, regarding carbon compounds, pathogens have highly consumed the essential structural compounds of cereals such as cellulose (amylopectin α-L-rhamnose, acid α-D-galacturonic acid, mannose), ß-glucans (glucose) and arabinoxylans (arabinose and xylose). These profiles were opposite to BCAs, which do not attack the plant directly, but have actually shown an ability to compete with pathogens, notably on soluble sugars known to be diverted by the pathogen (sucrose, glucose, etc.) [94], but also released naturally by the plant and used through symbiosis (e.g., mycorrhizae). Moreover, pathogens were generally good competitors for the plant’s nitrogen compounds, for many amino acids and forms of nitrogen that circulate in the plant (nitrate and nitrite). Such a diversity of plant sugar access mechanisms appears to reflect some of the differences in nutritional strategies between symbiotic and pathogenic fungi [95]. Other types of devices have been developed to measure nutrient competition but did not provide such a broad view of the compounds in competition [96,97]. Nevertheless, regarding Poly which privileges complex media, therefore, its average growth is lower in the presence of a single source of carbon or nitrogen. The study of nutritional competition for this BCA would be more adapted on a complex medium [64], where it has a higher specific growth rate.

### 4.7. Spatial Competition between BCAs and Pathogens and Potential Antimicrobial Metabolites Diffusion by BCAs

The space competition test provides a good estimation of the specific growth rates of microorganisms and the impact of the confrontation between these microorganisms on this capacity of progression. This mechanism of action is often associated with the production of degradation enzymes needed to colonize the medium (such as the chitinases). It is also associated with nutritional competition, but this link remains unclear. Indeed, the ability to transform various substrates to grow is not necessarily linked to the ability to colonize and spread over a surface, specific to the species. In order to better understand this mode of action, the spatial competition test could be improved to visualize the colonization of microorganisms on more complex matrices by using GUS-transformation [98,99] or by quantifying specific biomarkers by qPCR for example [100].

### 4.8. Impact of BCAs on Perithecia Production of F. graminearum

At the end of the life cycle of the pathogen, and in particular for *F. graminearum*, the pathogen produces ascospores enclosed in dark structures called perithecia. Plant infections by ascospores are known to contribute significantly to the severity of head blight *F. graminearum* epidemics [101], and residues of infected crops are the main source of inoculum for epidemics [102]. Despite this, little research has been conducted on the effect on perithecia of potential or commercially available BCAs for various stages of pathogen development. For many years, the traditional carrot agar medium of Klittich and Leslie [103] has been used to induce sexual reproduction by *Fusarium* spp. Some BCAs have shown their ability to reduce perithecia [104], notably *Clonostachys rosea* [105]. Repeated foliar applications of BCAs could increase its presence on debris buried in the soil and help reduce the inoculum of pathogens [106].

All BCAs tested reduced strongly the formation of perithecia. Concerning Myco, to our knowledge, this is the first report of direct observation of the action of *Streptomyces* spp. on the perithecia of *F. graminearum*. Xeda was also able to directly attack the formed mature perithecia. Coculture of *F. graminearum* perithecia with other *Trichoderma* species also showed decreases in pigment production, and the outer wall of the perithecia are deformed [107]. Poly severely limits the formation of perithecia and covers those that have been formed, mainly due to its strong expansion capacity. This species has already shown its ability to colonize and inhibit the germination of other survival structures, such as the sclerotia of the pathogen *S. sclerotiorum* [108]. However, this is the first time that it is being considered for the treatment of *F. graminearum* perithecia in soil, which would make it a BCA suitable for all phases of the pathogen’s life cycle (coupled with good colonization of aerial parts).

### 4.9. BCAs Global Behaviour against Mycotoxigenic Fusarium

Using this toolbox, different global behaviors could be highlighted in the three commercial BCAs studied against mycotoxigenic *F. graminearum* and *F. verticillioides* (Appendix A).

Overall, Myco’s biocontrol strategy is based on remote action via the synthesis of enzymes and antigerminative and antifungal compounds, but also a mixture of hydrolytic enzymes, that inhibit the progression of pathogens. It could have an effect before contact and during first closer interactions, through its mycophagous capacities. It has good nutritional competition capacity for wheat and maize compounds, suggesting a good establishment in the natural environment, but with limited capacity for spatial competition. Despite this, having been originally isolated from the soil, its use could be recommended against perithecia of *F. graminearum*.

Broadly, Xeda will be able to set up a large panel of antifungal mode of action with various targets (spores, mycelium or mycotoxins). Direct mechanisms such as the synthesis of anti-germinating compounds, the production of enzymes or volatile or nonvolatile antifungal compounds have been identified. It also acts as short-range antagonists, like many *Trichoderma* spp., via coiling, mycophagia or spatial competition, which support its necrotrophic mycoparasite character.

Globally, Poly has a very strong capacity for spatial competition and environmental colonization, which is a major advantage in the interaction with pathogens. Its strong capacity to inhibit pathogens occurs throughout the pathogen cycle via mycophagy mechanisms, stimulation of self-reproduction, limitation of growth and mycotoxin production, and perithecia control, with high efficiency, allowing it to be a major tool in the fight against mycotoxigenic fungi. However, it presents little aspect of specialization for the pathogens studied and is an aggressive parasite with a wide host range.

**Figure 12:**
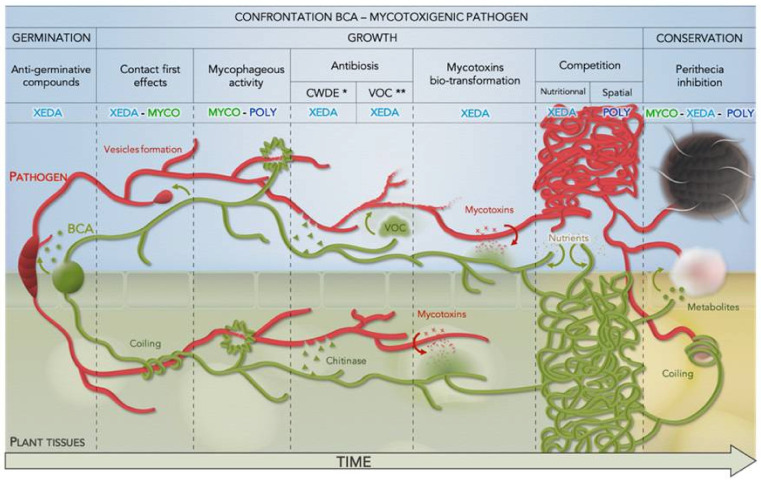
BCAs biocontrol mechanisms activity against pathogenic Fusarium over time. During confrontation between BCA and mycotoxigenic pathogen, a broad range of weapons can be used by BCAs, from the germination of pathogen, during growth phase, to conservation under perithecia structure. The best performing BCA(s) for each mechanism of action are noted as an indication in the third row of the figure. Depending on the BCAs considered, anti-germination compounds can be synthesized to inhibit the germination of pathogenic spores. The first contacts between microorganisms causes the formation of particular structures or deformations of pathogenic hyphae. BCAs can coil around the pathogenic mycelium and block their progression. Even in the absence of nutrients, they may be able to develop colonies around the active pathogen mycelium through mycophagous activities. BCAs, using a battery of secreted compounds such as cell wall degrading enzymes (*CWDEs) or antifungal volatile compounds (**VOCs), will limit the spread of the pathogen. BCAs are also able to directly degrade the mycotoxins produced or inhibit all or part of the biosynthesis pathway of these toxins in pathogens. Moreover, when the development of microorganisms is more advanced, competitions may occur. BCAs are able to attract the nutrients necessary for the growth of the pathogen, but also to colonize more space quickly to block the progression of the Fusarium. At the end of the life cycle of pathogens, BCAs can also prevent the formation of conservative structure, the perithecia, or interfere with them after they are formed.

As a result of this very varied and complementary set of experiences, a picture of the chronology of the interaction between BCAs and mycotoxigenic pathogens could be described (Figure 12).

## 5. Conclusions

Through dedicated experiments, this study allowed the identification of biocontrol mechanisms that occur in the interactions between BCAs and mycotoxigenic pathogens and highlighted a variety of strategies. Some BCAs have activated a wide variety of complementary mechanisms, while others have activated fewer modes of action during interaction but with greater individual effectiveness. Globally, *F. graminearum* is particularly sensitive to Xedavir activities such as the synthesis of anti-germinative and volatile anti-mycotoxigenic compounds, as well as mycophageous and chitinolytic activity. *F. verticillioides*, although affected by confrontations with Xedavir as well, is more sensitive to antifungal compounds secreted by Myco in the germination phases and volatile during growth but also in mycophagy tests. Poly has a more general biocontrol activity and acts mainly via spatial competition and mycophagy on both pathogens, but also specifically as for example on *F. graminearum* mycotoxins via volatile organic compounds. The investigation of the panoply of the modes of action deployed by the microorganisms could be more complete by considering an integrative approach combining this approach based on microbiology and biochemistry with other methods such as phytopathology and molecular biology, in upcoming studies.

## Figures and Tables

**Table 1:** MS/MS parameters for isotope labeled, internal standard (IS) (Romer Labs, Austria).

IS	Polarity	MRM	EC
U-(^13^C_15_)-DEOXYNIVALENOL	−	370.3 > 59.0	35
U-(^13^C_34_)-FUMONISIN B_1_	+	756.3 > 356.5	−47

**Table 2:** MS/MS parameters for mycotoxins. (Q) Quantification; (q) qualification.

Mycotoxin	Polarity	MRM Q	EC MRM Q	MRM q	EC MRM q	R^2^ Calibration Curve
DON	−	355.0 > 59.0	35	355.0 > 265.1	35	0.9998
15-AcDON	+	338.90 > 137.10	−19	338.90 > 297.20	−14	0.9988
3-AcDON	−	397.20 > 59.00	25	397.20 > 307.10	16	0.9988
FB_1_	−	722.35 > 334.5	−43	722.35 > 352.0	−44	0.9982
FB_2_	−	706.2 > 318.4	−37	355.0 > 265.1	−44	0,9980

**Figure A1:**
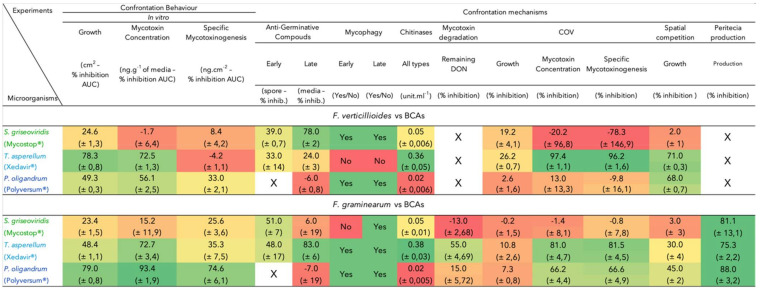
Overview table of BCAs behaviors and mechanisms of action during confrontation with *F. graminearum* and *F. verticillioides*. The columns indicate the experiments performed. The first columns (2, 3 and 4) present the results obtained in a previous work [21], the following columns (5–15) present the results obtained during this study, except for the results concerning the nutritional competition which are not quantitative. The lines indicate the microorganisms involved, lines 6–9: *F. verticillioides* vs. the different BCAs; lines 10–13: *F. graminearum* vs. the different BCAs. In each cell, the percentage of inhibition of the considered pathogenic factor is presented, or if necessary, the raw quantity considered (±standard deviation). The colors represent the level of inhibition in comparison to other BCAs (from the best performing, in green, to the worst performing, in red). Myco: Mycostop^®^, Xeda: Xedavir^®^, Poly: Polyversum^®^.

## Data Availability

The data presented in this study are available on request from the corresponding author.
